# The extent of windfarm infrastructures on recognised European blanket bogs

**DOI:** 10.1038/s41598-023-30752-3

**Published:** 2023-03-08

**Authors:** Guaduneth Chico, T. Clewer, N. G. Midgley, P. Gallego-Anex, P. Ramil-Rego, J. Ferreiro, E. Whayman, S. Goeckeritz, T. Stanton

**Affiliations:** 1 School of Animal, Rural and Environmental Sciences, Nottingham Trent University, Brackenhurst Campus, Southwell, NG25 0QF UK; 2 Instituto de Biodiversidade Agraria E Desenvolvemento Rural (IBADER), Universidade de Santiago de Compostela, Campus Terra, 27002 Lugo, Spain; 3 Department of Geography and Environment, Loughborough University, Loughborough, LE11 3TU UK

**Keywords:** Ecology, Environmental sciences

## Abstract

Peatland environments are the Earth’s largest terrestrial carbon store and have the potential to act as carbon sinks. However, the development of windfarms on peatlands is affecting their morphology, hydrology, ground-level climate conditions, carbon functions and vegetation, and long-term consequences still need to be assessed. Blanket bogs are a rare type of ombrotrophic peatland that are typical of oceanic areas with high precipitation and low temperatures. Their distribution has been mapped across Europe, where they are mainly located on hill summits where wind energy potential is higher, making them attractive sites for windfarm developments. The promotion of renewable energy is currently a priority given the environmental and economic drive to increase low-carbon energy production. Establishing windfarms on peatland in pursuit of greener energy, therefore, risks compromising and undermining the green-energy transition. Despite this, the extent of windfarm infrastructures on blanket bogs have not yet been reported at the European scale. This research reports the extent of windfarm infrastructures on recognised blanket bogs, with a geographical focus on Europe, where blanket bogs have been mapped systematically. Under the EU Habitats Directive (92/43/EEC), there are 36 European regions NUTS level 2 with recognised blanket bogs. Of these, 12 have windfarm developments, including 644 wind turbines, 253.4 km of vehicular access tracks and an affected area of 207.6 ha, mainly in Ireland and Scotland where the extent of blanket bogs is also higher. However, despite Spain having under 0.2% of Europe’s recognised blanket bogs area, this was the most affected country. In Scotland, a comparison of the recognised blanket bogs under the Habitats Directive (92/43/EEC) with blanket bogs recorded in national inventories indicates that the extent of windfarm developments was higher, with 1,063 wind turbines and 634.5 km of vehicular access tracks. Our results highlight the extent of windfarm developments on blanket bog habitat, both in areas where peatlands are broadly distributed across the landscape, and also in areas where this recognised habitat is particularly rare. There is a pressing need to assess the long-term impacts of windfarms on peatlands to ensure that efforts to meet energy targets result only in carbon sequestration, and do not jeopardise ecosystem services. Blanket bogs represent a particularly vulnerable habitat, the study of which should be prioritised updating national and international inventories to protect and restore this habitat.

## Introduction

Peatland environments are recognised as a potential carbon sink when in either restored or pristine condition^1^. This terrestrial habitat only covers 2.84% of Earth’s land surface^2^, but they represent over 25% of the total terrestrial carbon^3^. In Europe, only 5.2% of the land is covered by peatlands^2^, where Nordic countries concentred most of this habitat^4^. There are several peatland classifications based on environmental factors, such as climate, geomorphology and vegetation^5^; however, there is not a single factor classification system^6^. The hierarchical classification provides a method to study peatlands in relation to the scale of analysis with specific criterion for each level of analysis (e.g. nanotope, microtope, mesotope, macrotope and supertope)^7,8^. This enables the combination of vegetation, landforms, and hydrology to provide a better understanding of the relationships within peatlands environments^8^. There are two types of bogs based on the water sources and their topographical location; blanket bog and raised bog. The main geomorphological difference is whether they are covering the topography as a mantle (blanket bog) or if the formation is dominated by peat accumulation and a dome (raised bog) usually located in valleys or around water courses^9^. Blanket bogs are a rare ombrotrophic (rain-fed) type of peatland commonly found in hyperoceanic conditions with high precipitation (> 1000 mm yr^-1^), low temperature (< 15 °C) and low seasonal temperature variability^10^; although some exceptions are found in areas with lower precipitation (e.g. The Falkland Islands)^11^ highlighting the need of a global classification approach for this habitat. Globally, it has been estimated that blanket bogs cover around 10 million ha mainly in the Northern Hemisphere^10^. In Europe, the most important and extensive blanket bogs are found in the British Isles with some occurrence in Norway, France, Austria, Sweden, Spain and Portugal (Azores Islands)^12^. Blanket bogs usually cover large extensions of the landscape, although they could be covering small areas in certain locations, mainly because of topographical limitations (e.g. high slope)^9,13^. Peat depth can vary from a few centimetres to several metres depending on the climatic conditions and topography^9^.

**Figure 1:**
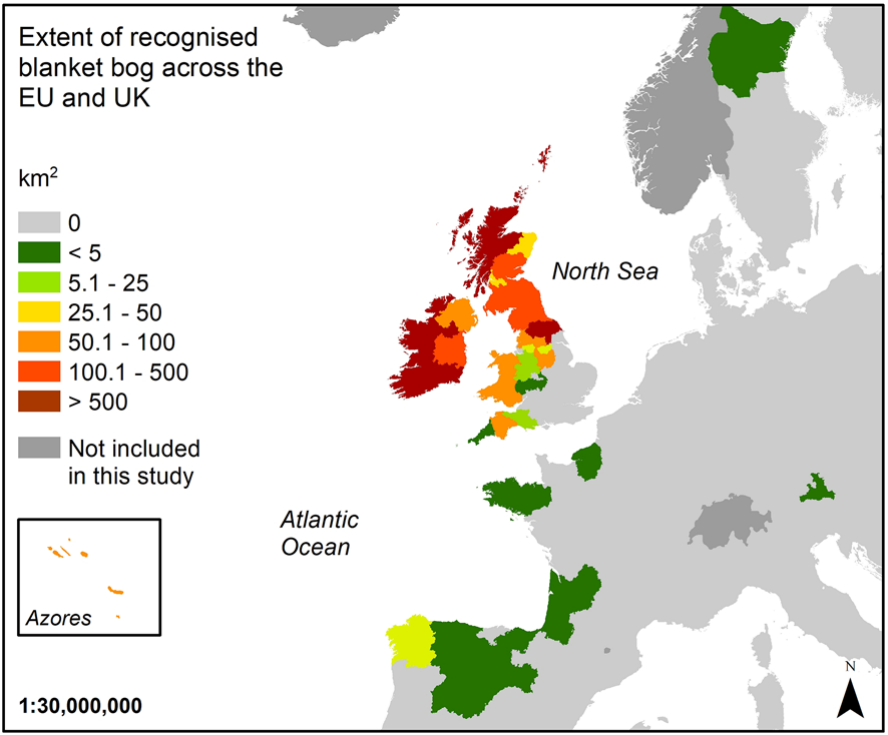
Total extent of the recognised blanket bog by European regions NUTS level 2 based on the Habitats Directive (92/43/EEC) including the last update from the UK before Brexit. Map created in ArcGIS 10.8.1.

Since 1992, in the European Union (EU) and the United Kingdom of Great Britain and Northern Ireland (UK), the Habitats Directive (92/43/EEC) has recognised blanket bogs (habitat code: 7130) as a terrestrial habitat, and a priority habitat if active (Fig. 1)^14^. This habitat has also been included in the European Red List of habitats assessed as near threatened because the strong decline in the quality of the habitat over the last 50 years^15^. This legislation has required each member state to since report on the conservation status of this habitat every six years and, when necessary, encouraged and implemented restoration actions to improve the quality and conservation status of the habitat. When reporting the conservation status, a detailed understanding of the extent of this habitat is needed. However, several unrecognised blanket bogs have been described across the EU recently, highlighting the lack of understanding and consequently adequate protection of this important habitat^16,17^. In addition, some blanket bogs are recognised as special areas of interest and also included under Natura 2000, providing additional legal protection for key habitats.

Notwithstanding the extensive peatland restoration projects (e.g. LIFE programme) and their positive implications for peatland ecosystem services^18^, a large proportion of these blanket bogs are currently in an unfavourable condition^19^. Blanket bogs with favourable status are only found in France, Sweden and Portugal^20^, where they represent less than 2% of the total extent of recognised blanket bogs across the EU and the UK^19^. Pressures such as peat extraction for fuel and horticulture, forestry, overgrazing, drainage, burning for recreational activities^21^ and anthropogenic infrastructures, have affected the stability of blanket bog habitats for centuries. Current threats frequently included in Habitat Directive conservation status reports for blanket bogs are variable across Europe (Table 1), although agriculture, drainage, and intensive grazing are the most common issues^19^. However, more recently, windfarm developments^17,22,23^ have been highlighted as a concerning pressure for this habitat having impacts on peatland hydrology^17,23^, ground-level climatic conditions^24^ and habitat biodiversity, contributing to vegetation changes and habitat transition^25^. This pressure not only affects the areas where turbines are installed, but also where associated infrastructures, such as tracks, sub-stations and associated facilities are built^23^. Windfarm infrastructures have an impact on the peatland surface affecting the hydrological units^23,26^, as well as promoting vegetation changes and habitat loss^25^.

**Table 1:** Pressures and threats on recognised blanket bogs across the EU and UK according to the last habitat assessments^19^.

Country		Wind and Energy	Intensive grazing	Agriculture	Peat extraction/cutting	Invasive species	Forestry	Drainage	Abiotic pressures	Tourism /Sport/Recreation	Infrastructure/Mining	Air pollution	Climate Change	Burning	Management of game	Other
Spain	Pressure	M	M	M	H	M								M		
	Threat	M	M	M	H			M					M	M		
Republic of Ireland	Pressure	M	H	H	H		H	M	M					H		
	Threat	M	H	H	H		H	M	M				M	H		
United Kingdom	Pressure	M	H		M	M		H				H	M	H	H	M
	Threat	M	H		M	M		H				H	M	H	H	M
Portugal	Pressure		H	H			H	H	M	M						M
	Threat		H	H			H	H	M	M						M
France	Pressure															M
	Threat			M				M	M			H	H			
Austria	Pressure		H					M		M		M	M			
	Threat		H					M		M		M	M			

Sweden has not reported this information in their last habitat assessment. H = High, M = Medium.

The Directive EU 2018/2001 on the promotion of the use of energy from renewable resources aims to promote the use of renewable energy across the EU^27^ based on Article 194 of the Treaty on Functioning of the European Union, requiring member states to promote energy efficiency and saving^28^. By 2030, at least the 32% of the energy consumed across the EU should be renewable. In 2020, 36% of the EU renewable energy was produced by windfarms, demonstrating the importance of this technology in reducing our carbon emissions^29^. Although the need to expand international renewable energy capacities is clear, it is important that the pursuit of legislative energy requirements and targets does not compromise the aims of alternative environmental legislation. Therefore, a legislative conflict exists between the Habitats Directive and Renewable Energy Directive that must be acknowledged and addressed. Both directives aim to reduce carbon emissions by restoring carbon sinks and promoting renewable energy transitions and, though windfarm development is permitted on Natura 2000 sites or recognised blanket bogs under the Habitats Directive, if they negatively affect the integrity of the site and the impacts cannot be mitigated by additional measures, authorisation for the development should not be granted^29^. Noting the lack of understanding regarding the long-term impacts on the peatland carbon sink function and the fact that windfarms installed on non-degraded peatland would not result in a net long-term carbon benefit^30^, windfarm developments need to be carefully assessed.

There is an urgent need to assess the current extent of blanket bog windfarm developments to minimise the impact on this protected and recognised habitat, and to inform site selection to prevent future degradation of natural carbon storage and carbon sinks in the pursuit of green energy.

Due to the recent European Union policy changes that promote renewable energy and aim for carbon neutrality by 2050^31^, and the identification of blanket bogs that have not previously been mapped^26^, there is a need to report the current extent of windfarm developments on blanket bogs and to assess further the implications and long-term impacts that these developments have on peatland ecosystems.

This research aims to: (1) assess for the first time the current known extent of windfarm developments on recognised blanket bogs across the EU and UK under the Habitats Directive (92/43/EEC) at European regions level; (2) quantify the extent of windfarm infrastructures on recognised blanket bogs at county level by country; and (3) compare the extent of windfarm developments on recognised blanket bogs under the Habitats Directive (92/43/EEC) with other blanket bogs data available (e.g. Scotland).

## Results

### European scale

**Figure 2:**
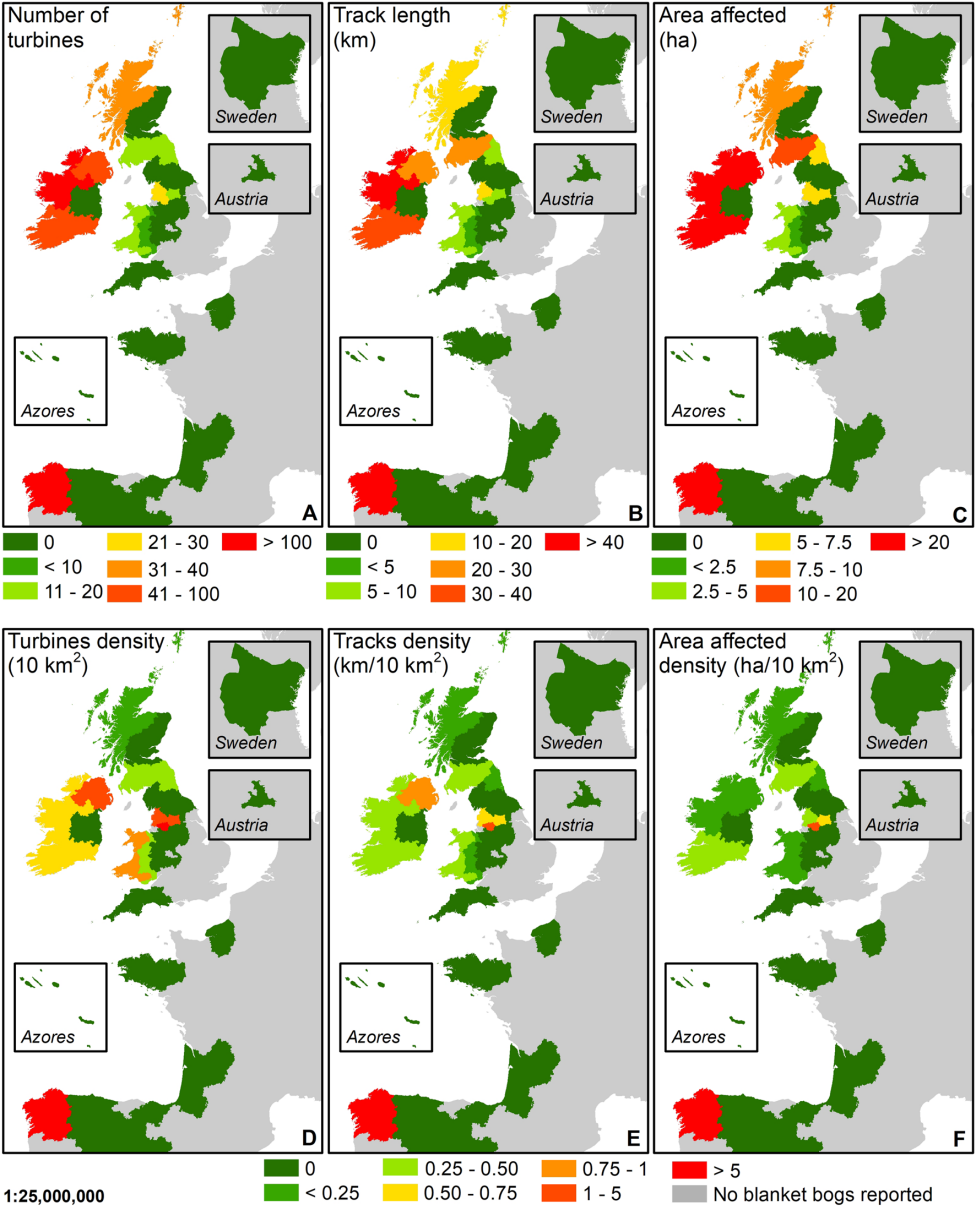
The extent of windfarm developments across the EU and UK on blanket bog habitat 7130 based on the 2013–2018 conservation status report^19^ prior to Brexit by European regions NUTS level 2^32^. *Note: Boundaries of the regions have not been added to improve legibility. The political geographical regions do not represent the extent of blanket bogs.* (**A**) Total turbines on blanket bog habitat 7130. (**B**) Tracks length on blanket bog habitat 7130. (**C**) Total area affected by windfarm developments on blanket bog habitat 7130. (**D**) Density of turbines per 10 km^2^ of blanket bog habitat 7130 by European regions. (**E**) Density of tracks per 10 km^2^ of blanket bog habitat 7130 by European regions. (**F**) Density of area affected by windfarm developments per 10 km^2^ of blanket bog habitat 7130 by European regions. *Datasets for blanket bog habitat 7130*: Austria, Portugal, Sweden and France^19^, Scotland^33^; England^34^; Wales^35^; Republic of Ireland^36^ and Spain^37^. *Dataset for peatlands (only priority habitat)*: Northern Ireland^38^. The location of mapped regions in the Azores, Austria, Sweden, and the full extent in Spain is shown in Fig. A (Supplementary materials). Map created in ArcGIS 10.8.1.

This assessment reports the extent of windfarm developments, including the number of wind turbines (Fig. 2A/D), the length of vehicular tracks (Fig. 2B/E), and the total area affected by the windfarm developments (Fig. 2C/F) across European blanket bogs designated as blanket bog habitat 7130 under the Habitats Directive (92/43/EEC). A total of 644 wind turbines, 253.4 km of access vehicular tracks and 207.6 ha of area affected by windfarm developments were found on the national inventories of recognised blanket bogs under the Habitats Directive. Due to the limitations of using aerial imagery to digitise windfarm infrastructures, it was not possible to map all the associated facilities when not visible, such as power lines. This assessment is, therefore, a likely underrepresentation of the extent of all windfarm developments on blanket bogs.

At the country scale, Sweden, Austria and Portugal did not have any windfarm developments on recognised blanket bog habitat 7130 (Fig. 2); however, as highlighted previously, these countries represent a small proportion of the total designated blanket bog extent^19^. Despite Spain representing under 0.2% of the total area of recognised blanket bogs across the EU and the UK^19^, it had the highest density of wind turbines with > 1 turbine for each 10 km^2^ of recognised blanket bog, comparable only with the north of England (Greater Manchester) in the UK (Fig. 2D). When exploring the density of vehicular tracks and the area affected (Fig. 2E,F), northwest Spain (Galicia) had the largest density of both variables, wind turbines (8.1 turbines/10 km^2^) and vehicular tracks (2.3 km/10 km^2^).

The Republic of Ireland is the country with the largest extent of windfarm infrastructures installed on recognised blanket bog (Fig. 2A–C); however, densities are lower because of the large area of recognised blanket bog in this country (Fig. 2D–F). A comparable situation is found in the north and south of Scotland, in the European regions of Southern Scotland and Highlands and Islands (Fig. 2D–F). Finally, Northern Ireland also has installed many windfarm developments on recognised blanket bogs; though, the total area affected by the windfarm infrastructures (0.2 km^2^) is smaller when compared to the total extent of recognised blanket bog (219.4 km^2^, Fig. 1).

Across Europe, the designation of blanket bog habitat 7130 under the Habitats Directive does not automatically provide protection; although there is an obligation for EU member states to implement conservation actions to improve this habitat. Windfarm developments across the Natura 2000 network are rare (e.g. Scotland, Table 2), but do occur (e.g. Spain and Ireland, Table 2). This situation occurs when a windfarm development does not affect the integrity of the protected site^29^.

**Table 2:** Windfarm developments on recognised blanket bog habitat 7130 compared with recognised blanket bog habitat within a Natura 2000 site.

Country	Recognised as blanket bog habitat 7130			Recognised as blanket bog habitat 7130 and protected under Natura 2000 network		
	Turbines	Track length (km)	Track area (ha)	Turbines	Track length (km)	Track area (ha)
Spain	252	70.7	46.3	233	65.9	43.7
Republic of Ireland	186	92.2	82.9	24	12.1	9.3
England	72	36.4	28.1	0	0	0
Wales	24	7.8	5.4	0	0.07	0.06
Scotland	55	26.6	25.5	0	1.4	0.9
Northern Ireland	430	203.2	213.9	0	0	0

No relationship between the extent of recognised blanket bog and number of turbines (*p* = 0.30), length of tracks (*p* = 0.40) and total affected area (*p* = 0.46) was found.

### Republic of Ireland and Northern Ireland

**Figure 3:**
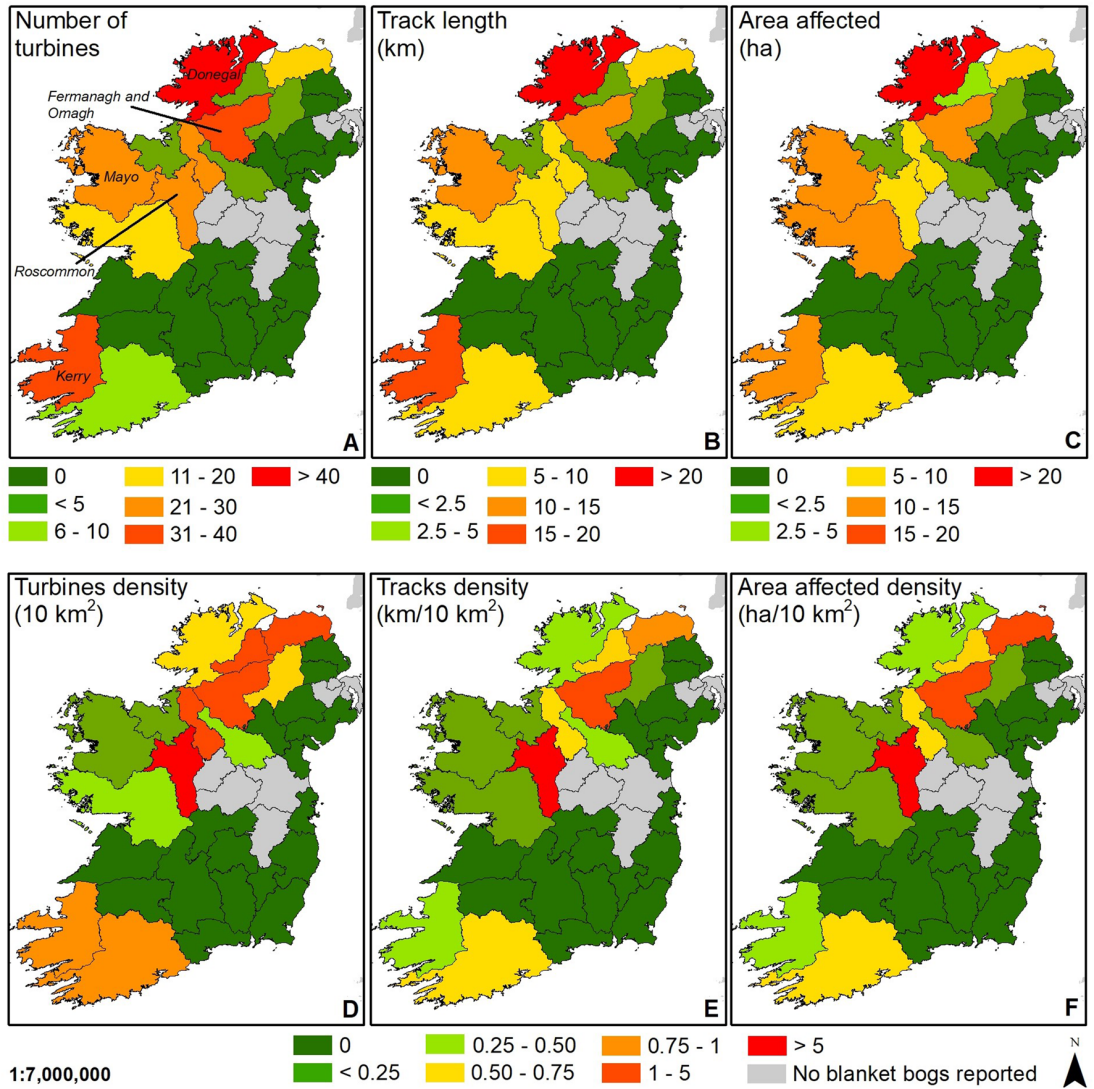
The extent of windfarm developments across the island of Ireland on blanket bog habitat 7130 at county level for the Republic of Ireland and intact or pool complex after blanket bog visual assessment for peatlands on Northern Ireland. (**A**) Total turbines on blanket bog habitat by county. (**B**) Tracks length on blanket bog habitat by county. (**C**) Total area affected by wind farm developments on blanket bog habitat by county. (**D**) Density of turbines by 10 km^2^ of blanket bog habitat by county. (**E**) Density of tracks by 10 km^2^ of blanket bog by county. (**F**) Density of area affected by wind farm developments by 10 km^2^ of blanket bog habitat by county. *Dataset for blanket bog habitat 7130*: Ireland^36^. *Dataset for peatlands*: Northern Ireland^38^. Map created in ArcGIS 10.8.1.

In the British Isles, blanket bogs are common and the island of Ireland (including Republic of Ireland and Northern Ireland) represents one of the most extensive hotspots for the conflict between windfarm developments and recognised blanket bog habitat. Within the island of Ireland, the northwest (Co. Donegal) is the most affected by windfarm developments by total extent (Fig. 3A–C). When assessing densities (Fig. 3D–F), this area concentrates a high proportion of the total extent of the island’s recognised blanket bogs (> 20%) meaning a lower density of windfarm infrastructures. Similar conclusions can be drawn in some areas of the east of the Republic of Ireland (Co. Kerry and Co. Mayo) and southwest of Northern Ireland (Co. Fermanagh and Omagh; Fig. 3). Together, these locations represent almost 60% of Ireland’s recognised blanket bogs. Beyond these four areas, blanket bogs in central Ireland (Co. Roscommon) had a particularly high density of windfarm infrastructures (Fig. 3D–F). Despite the small extent of recognised blanket bogs across Co. Roscommon (8.5 km^2^), extensive windfarm infrastructure has been developed across blanket bog in the county.

In Northern Ireland, although the current assessment has shown an important number of windfarm infrastructures on blanket bogs (Fig. 3), the total extent of blanket bog habitat 7130 could be much greater due to the lack of detailed data as it is stated to only include the most intact blanket bogs in our analysis. Further analysis is needed to fully assess the extent of the issue, which appears to be larger than reported. In fact, the most recent conservation status report (2013–2018)^19^ for blanket bogs reported a total area of 1400 km^2^, while the extent assessed in this research is only 219 km^2^. When assessing the full extent of priority habitat peatlands across Northern Ireland^38^, increases were observed in the number of wind turbines (+ 376, + 796%), the length of tracks (+ 183.2 km, + 1,021%), and the area affected by windfarm infrastructure (+ 1.92 km^2^, + 972%).

### Scotland

**Figure 4:**
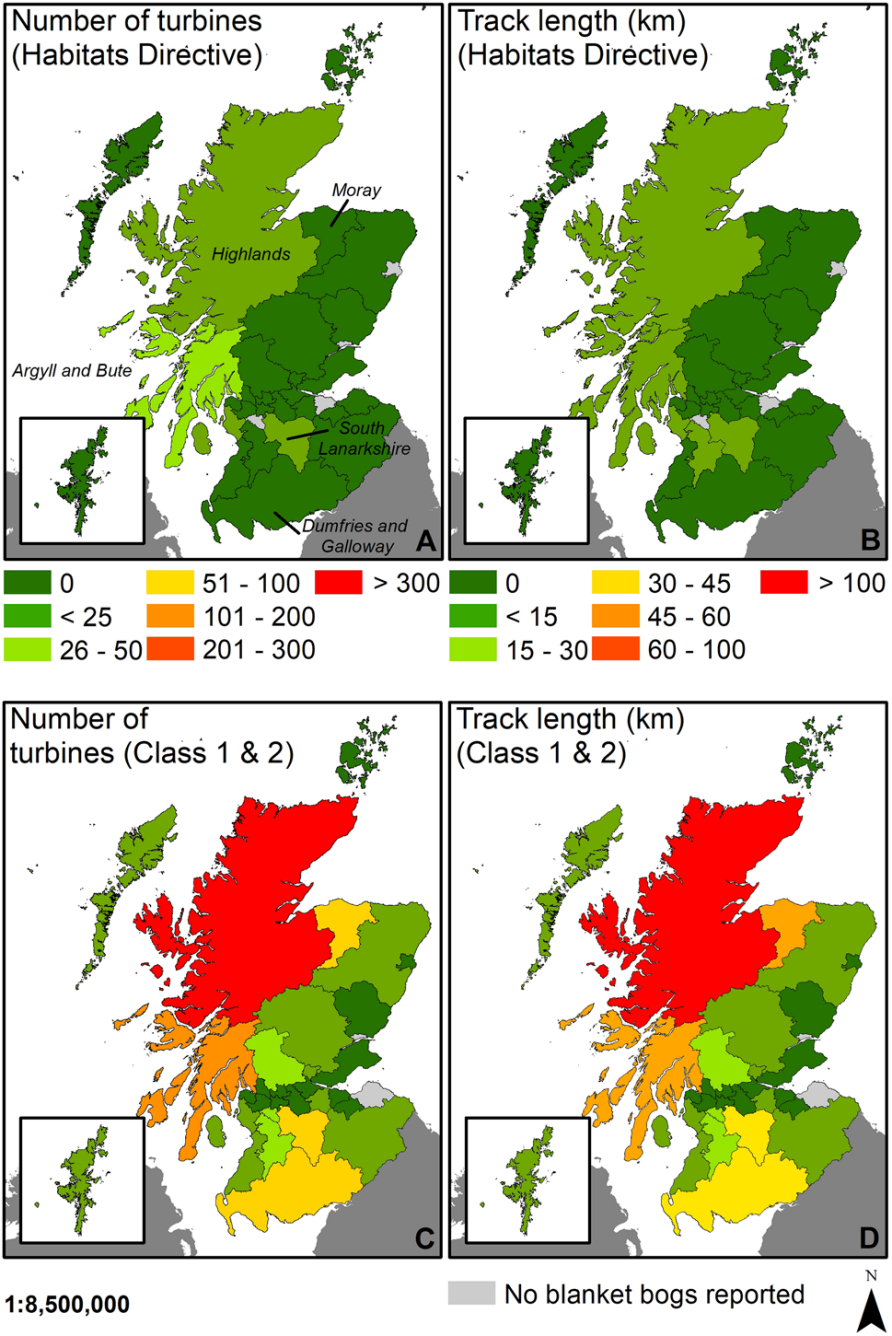
The extent of windfarm developments in Scotland by county. (**A**) Total turbines on blanket bog habitat by county. (**B**) Tracks length on blanket bog habitat by county. (**C**) Total turbines on Class 1 & Class 2 peat soils by country. (**D**) Tracks length on Class 1 & Class 2 peat soils by county. *Dataset for blanket bog habitat 7130*: Scotland^33^. Dataset for Class 1 and Class 2 peat soils^39^. Map created in ArcGIS 10.8.1.

North and South of Scotland (Southern Scotland, and Highlands and Islands European regions) are the most affected by windfarm developments on recognised blanket bogs under the Habitats Directive (92/43/EEC) within Scotland (Fig. 2); however, when assessing the country at county scale, Argyll and Bute county located on the west coast of Scotland, contains the majority of the windfarms and tracks in Scotland (Fig. 4A, B). Although recognised blanket bogs at the European scale in Scotland showed a low density of windfarm infrastructures, when assessing the number of turbines, length of tracks, and total area affected it showed higher numbers than other European regions (Fig. 2). However, the total extent of recognised blanket bog is an underestimation of the total extent of this habitat when compared with the national classification of peat soils. Since 2016, Scotland classified carbon and peatland classes across the country based on presence of carbon-rich soils, deep peat and priority peatland habitat for each area, at a coarse scale^39^. The government gave an indicative value of the habitat and whether the habitat is a priority peatland. Of the 8 classes, class 1 and 2 are both considered ‘Nationally important carbon-rich soils, deep peat and priority peatland habitat’^39^. These classes are differentiated by their likely (Class 1) and potential (Class 2) conservation value and restoration potential, and therefore, when assessing the extent of windfarm developments, these areas need to be considered to fully assess the extent of the conflict between windfarms and peatlands in Scotland.

A total of 1,063 wind turbines and 634.5 km of tracks have been developed on priority peatland Class 1 and 2 across Scotland in comparison with 51 wind turbines and 22.6 km of tracks installed on recognised blanket bog under the Habitats Directive (Fig. 4). The most affected area is the north of the country (Highlands) representing over 46% of the wind turbines in Scotland (Fig. 4C, D). Other areas of Scotland such as the west coast (Argyll and Bute county), south (Dumfries and Galloway, and South Lanarkshire counties) and northwest (Moray county) also had an important number of windfarm infrastructures on priority peatland habitat according to the national inventory (Fig. 4C, D). It is worth noting that the Class 1 and 2 peatlands could also include other peatland priority habitats, such as active raised bogs. When windfarm developments were assessed on active raised bogs under the Habitats Directive^33^, none were found indicating that the total reported is only on blanket bog habitat.

### England and Wales

**Figure 5:**
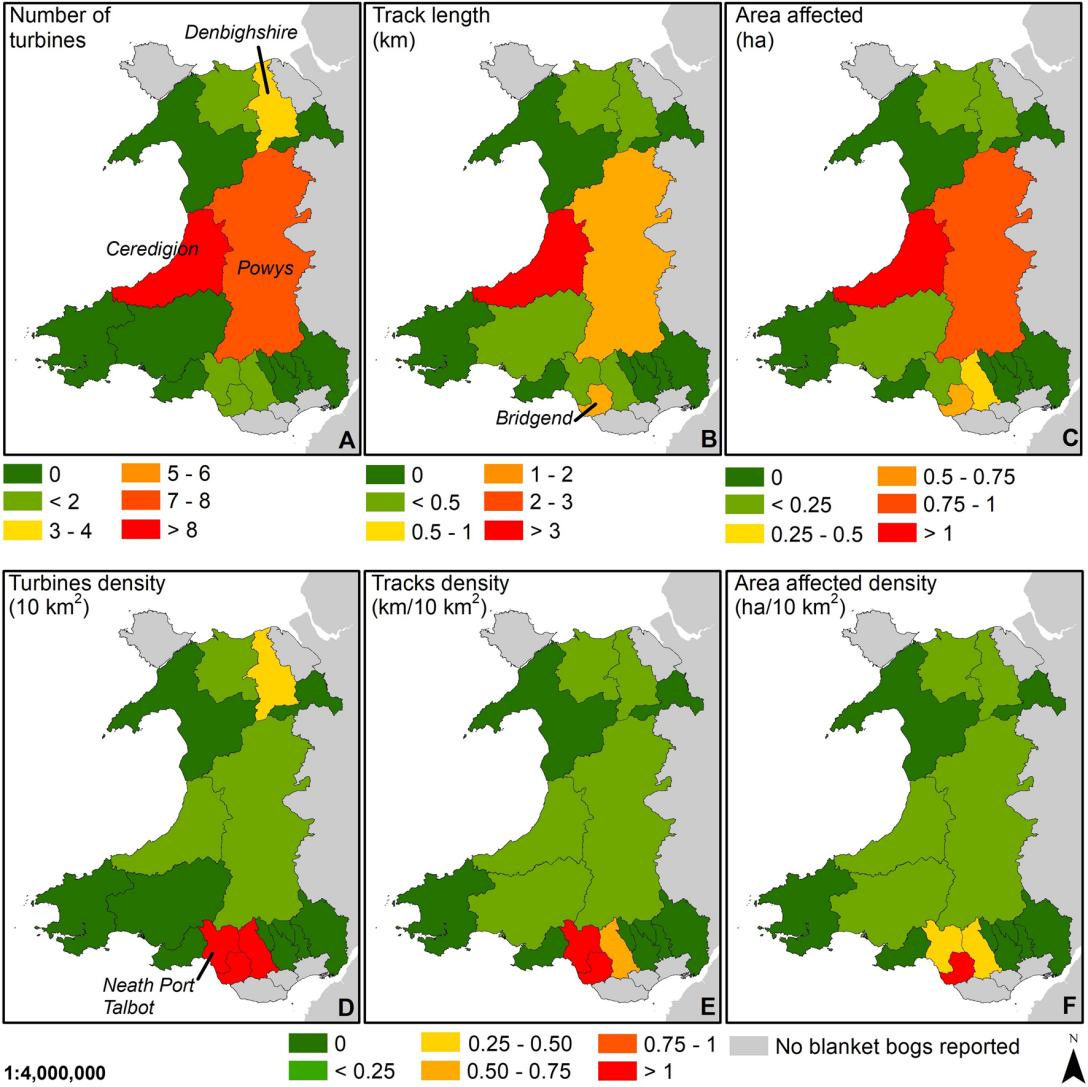
The extent of windfarm developments in Wales by county. (**A**) Total turbines on blanket bog habitat by county. (**B**) Tracks length on blanket bog habitat by county. (**C**) Total area affected by wind farm developments on blanket bog habitat by county. (**D**) Density of turbines by 10 km^2^ of blanket bog habitat by county. (**E**) Density of tracks by 10 km^2^ of blanket bog by county. (**F**) Density of area affected by wind farm developments by 10 km^2^ of blanket bog habitat by county. *Datasets for blanket bog habitat 7130*: Wales^35^. Map created in ArcGIS 10.8.1.

As in other countries, when assessing the extent of windfarm developments at county level, the issue seems to be concentred in specific counties rather than across all the European region. In Wales, the counties of Ceredigion and Powys showed the higher number of windfarm developments (Fig. 5A–C), but densities are higher in South Wales (Fig. 5D–F) where the extent of blanket bogs is lower (Fig. 1). In England, Greater Manchester was still the main region with the higher density of windfarm developments (Fig. 2D–F).

### Spain

Galicia, an administrative region located in the northwest corner of Spain, currently holds the most extensive recognised blanket bog area in Spain. Despite the region only containing 31.2 km^2^ of recognised blanket bog (Fig. 1), this region is the most affected by windfarm developments when assessing the density and total number of turbines and tracks (Fig. 2). Windfarm infrastructures have also been reported across other blanket bogs in the Cantabrian Mountains^17^, but since the blanket bogs have not been yet recognised under Habitats Directive, they have not been included in this research.

## Discussion

When studying windfarm developments at the European region scale, the high densities of windfarm developments on blanket bog in Galicia and Greater Manchester (north England) are influenced by the total extent of the recognised blanket bog which is lower in Spain (31.2 km^2^ total extent) and in Greater Manchester (40.8 km^2^ total extent) in comparison to other regions (Fig. 1), although no relationship between the total extent of recognised blanket bog and the windfarm developments (wind turbines, tracks and total affected area) was found. Although the rest of the European regions across Spain showed lower densities of windfarm infrastructures (Fig. 2), the total extent of recognised blanket bogs across those regions was under 1 km^2^ (Fig. 1) meaning that the majority of recognised Spanish blanket bogs could be under threat due to their small size and the potential impact of windfarm infrastructures, if installed. In addition to this, previously unrecognised Spanish blanket bogs that have now been reported^17^ that could also be under pressure as the lack of formal recognition and protection leaves this habitat exposed to a range of anthropogenic activities, including windfarm developments. In fact, some examples of blanket bogs with extensive damage have been identified and reported in Galicia^25^, and more recently in Cantabrian blanket bogs^17^.

Spanish unmapped areas of blanket bog at the edge-of-range of this habitat in the south of Europe are, therefore, particularly at risk from windfarm developments, and may disappear before their extent and importance can be defined^40^. Currently, new renewable energy regulations have been developed as a result of the climate emergency, and several windfarm developments have been proposed in ecologically sensitive areas, where blanket bogs have been reported (e.g. Sierra del Escudo, Spain) increasing the pressure on this habitat. Spanish blanket bogs also have specific characteristics, such as their small size as a consequence of the topographical limitations (e.g. slope) for their development^26^, meaning that they usually only cover the hill summits, where wind energy potential is at its greatest. Since blanket bogs are small and the windfarm development may cover all of the hill summit for their installation, many blanket bogs will be irrevocably damaged^40^.

Most of the Galician blanket bogs were protected in 1999, under the Natura 2000 network and were declared as Special Area of Conservation (SAC) in 2014. However, between 1999 and 2012, Galician blanket bogs underwent severe and significant alterations in the peatland surface as a consequence of the large number of windfarm developments^41^ that were established during the period (Table A—Supplementary information), even when the site was incorporated into the Natura 2000 network (Table B—Supplementary information). Despite available scientific evidence that showed the potential environmental risks for these vulnerable ecosystems, windfarms were installed in what this work found to be the most extensive windfarm infrastructures across recognised European blanket bogs (Fig. 2).

The incomplete current understanding of the extent of Spanish blanket bogs highlights the need to improve the completeness and representativeness of their current records across the Spanish Atlantic biogeographical region to include, within Natura 2000, a sufficient cover of their occupied area, in proportion to the representation of this natural habitat type in the Member state, for which it could therefore be concluded that the network is complete. Due to the increasing evidence highlighting how important the transitional areas are within the blanket bog complex^42^, other peatland types and wet heaths should be also considered when recognising and protecting blanket bogs. Mapping unrecorded blanket bogs must be a priority to fully understand the geographical and climatic range of this habitat, and obligatory protection under the Habitats Directive (92/43/EEC) is key to protecting the southern edge-of-range of this habitat.

In addition to the lack of protection and updated inventories, the priority status included in the Habitats Directive, key to promoting their protection and restoration, is only for active blanket bogs, excluding other degraded blanket bogs with the potential to be active (carbon sinks), if they are restored. An approach similar to that of Scotland, where degraded blanket bogs are included^33,39^, could promote blanket bog restoration across Europe and improve the protection of this natural carbon storage.

Many countries have also misinterpreted the active status of the blanket bog meaning that it is difficult to define whether the recognised blanket bog habitat is classed as a priority or not. Some countries, such as the Republic of Ireland, have classified as 7130 only active blanket bogs^36^, meaning that degraded blanket bogs lack appropriate classification and incorrectly applying the Habitat Directive designation as not all blanket bogs are included. The priority status is given when the habitat is particularly vulnerable or unique to the EU and necessitates additional measures for their protection and surveillance; however, whilst some blanket bogs may not act currently as carbon sinks, they still contain large amounts of carbon, and when restored they can recover their carbon sink function^1^, and then act to mitigate climate change.

The issue of windfarm developments across the Republic of Ireland has been previously reported using a peat map^43^. However, despite researchers highlighting the importance of excluding vulnerable peatland ecosystems in future developments^44^, new areas of windfarms have been built affecting further recognised blanket bogs. At least 79 wind turbines have been installed in the Republic of Ireland since 2008 on recognised blanket bogs (Table A—Supplementary information) representing the 9.8% of the total onshore turbines installed in the country (Table 3), highlighting the importance of this conflict. The contribution of wind energy production to electricity supply was predicted to be up to 30% by 2020^44^. In 2020, wind energy consumed in the Republic of Ireland represented 36%^45^. This represented an average annual increase of wind energy consumption of 16.9%^45^ between 2005 and 2020, which may explain part of the increase of 42% in wind turbines since 2008 (Table A—Supplementary information).

**Table 3:** Total % of turbines on blanket bog (recognised/national inventories) in relation with the total turbines installed by country*.*

Country	Total number of turbines	Turbines on blanket bog	% of turbines on blanket bog
Spain	21,574^51^	252	1.2
Republic of Ireland	1896**^47^	186	9.8
England	2138*^52^	72	3.4
Wales	1177*^52^	24	2
Scotland	6322*^52^	55 (protected)	0.9
		1,063 (Class 1 & 2)	16.8
Northern Ireland	1950*^52^	54 (only intact blanket bogs)	2.8
		430 (all priority peatlands)	22.1

*Include wind turbines consented, operational and under construction. **Include proposed, constructed and under construction.

Across Europe, several governments have developed climate action plans that over the next decade promote renewable energies to reduce carbon emissions. The government of the Republic of Ireland is aiming to generate up to 80% of electricity from renewable energy by 2030, providing support for onshore windfarm developments with an increase of up to 32% of the renewable energy production by 2030, but with a favourable preference for offshore wind energy production (up to 52% of the renewable energy production)^46^. This may help to reduce the conflict between blanket bogs and windfarm developments. Currently, windfarm annual energy production on blanket bogs accounts for 263.4 MW, 6.1% of the total production of wind energy in the Republic of Ireland^47^.

The promotion of onshore wind energy production^46^ and the lack of protection of the full extent of blanket bogs are also threats that need to be considered in the Republic of Ireland. In 2008, a peat map was published showing the distribution of blanket bogs and raised bogs across the Republic of Ireland^43^. However, the inventory of current recognised blanket bogs under the Habitats Directive does not cover the full extent reported in this research^43^. While the total extent of recognised blanket bogs under the Habitats Directive 92/43/ECC reported a total of 3621 km^2^ of blanket bogs^36^, the real extent of blanket bogs across the country could be up to 2.5 times more (9202 km^2^)^43^, highlighting the lack of protection and the potential further increase of the windfarms and peatlands conflict in the Republic of Ireland as it happens in Spain and Scotland.

The lack of recognition of blanket bog habitat in combination with the promotion of wind energy production across the island of Ireland could affect further areas of blanket bog, increasing the degradation of blanket bogs. An urgent review of inventories needs to be promoted in both countries, the Republic of Ireland and Northern Ireland, to fully assess the impact of the extensive areas of windfarms across the whole island.

In Scotland, the pressure of windfarm developments on blanket bogs is also evident, where the Scottish Planning Policy considers classes 1 and 2 as areas of significant protection; although, windfarm developments may be possible under some circumstances^48^ as is permitted under the Habitats Directive across the EU^29^. However, to assess the impacts of windfarms on peatlands in a consistent way and evaluate the environmental impact of potential new developments on carbon-rich soils, a carbon calculator has been developed by the Scottish Government^49^. The carbon calculator allows users to estimate the carbon savings of windfarms installed on peatlands, although they highlight the importance of long-term management in relation to the final net carbon calculation^49^. Nonetheless, installing windfarms on non-degraded peatlands has been reported as unlikely to reduce carbon emissions even when the management has been considered carefully and it should be avoided ^30^. Therefore, peatlands under classes 1 and 2 considered by the Scottish government as a priority should be excluded from any windfarm developments (currently representing over 16% of onshore turbines, Table 3); especially considering the current policy of increasing onshore windfarms in Scotland^50^. Long-term research is needed to fully assess the impacts before new windfarm developments are installed.

The difference between the recognised blanket bogs included in the EU Habitats Directive and the Scottish national inventory highlights the importance of updating and defining the complete extent of blanket bogs to facilitate their protection and restoration.

In this novel research, the extent of windfarm developments across all recognised European blanket bogs under the Habitats Directive have been assessed. Large extents of blanket bogs have already been damaged, concentrated in the edge-of-range of this habitat and directly affecting hundreds of hectares of blanket bog across the rest of Europe. The full potential long-term damage to the habitat functionality is still unclear, but scientific evidence supports the negative impacts of windfarm developments on this critical habitat. European blanket bogs need further scientific evidence to demonstrate the real benefit of incentivising the reduction of carbon emissions by installing onshore windfarm infrastructures on peatlands which are causing the degradation of the most important long-term natural carbon sink and storage ecosystems. A strategic restoration plan and appropriate relevant legislation would be beneficial to promote the safeguarding of blanket bogs in the UK after Brexit. An urgent revision and compliance of the legislation regarding the protection of blanket bogs needs to be implemented, especially under the current trend of promotion and increasing legislation on renewable energy to reduce carbon emissions. An improvement of the national inventories across the EU and UK protected area networks is critical to implement the recognition, protection, and restoration of this habitat, in order to guarantee its favourable conservation status and its function as a long-term carbon sink to mitigate climate change.

## Methods

### Geographical context

To assess the extent of windfarm infrastructures across the EU and the UK, two geographical levels have been studied: European region NUTS Level 2 and County level for England, Scotland, Northern Ireland and Republic of Ireland. Due to the small size of some blanket bog areas and the large scale of the study, data extracted from this analysis has been associated with each geographical level for cartographical representation. Therefore, the total extent of the geographical regions does not represent the total extent of blanket bogs.

### Data collection and cleaning

All data related to recognised blanket bogs areas were collected from official governmental websites (Table 4). When all habitats (e.g. all peatlands) were included in the shapefile, blanket bogs were selected in ArcGIS 10.8.1 and exported as a new vector shapefile dataset to only assess blanket bogs when possible.

**Table 4:** Data source for each blanket bog area by country.

Country	Dataset	Data source
Spain	Recognised blanket bog	IBADER—University of Santiago de Compostela^37^
Republic of Ireland	Blanket bog (Active)	National Parks and Wildlife Service^36^
England	Priority Habitat Inventory	Natural England^34^
Wales	Priority Habitats	Welsh Government^35^
Scotland	Habitat Map of Scotland	NatureScot^33^
	Carbon and Peatland map	NatureScot^39^
Northern Ireland	Priority Habitat—Peatlands	Northern Ireland Environment Agency^38^
Austria, Portugal, Sweden and France	Blanket bogs (*if active bog)*	European Environment Agency^19^
All other EU countries	No blanket bog habitat reported	

*For Austria, Portugal, Sweden and France, there is no specific dataset for blanket bog areas when the study was completed. The total extent of Nature 2000 sites where blanket bog has been reported were assessed to determine if windfarm infrastructures were present on the site.

Spain, England and Republic of Ireland did not require additional processing since all vector data obtained was already reported as blanket bog habitat 7130. For Northern Ireland, all priority peatlands were included in the initial vector data file; consequently, a selection of only peatlands defined as ‘intact’ or ‘pool complex’ were selected for the main analysis followed by a visual assessment based on topographical characteristics of blanket bogs to exclude potential raised bogs (e.g. valleys or river basin areas). Scotland and Wales blanket bogs habitat were reported along with the rest of the Habitats Directive classes; therefore, blanket bogs were selected and exported using ArcGIS 10.8.1 for analysis. France, Sweden, Austria and Portugal did not have any available data, but since blanket bog extent is very limited, this was assessed using the blanket bog database from the EU in Google Earth Pro assessing all the protected areas from Nature 2000 for windfarm infrastructures. In Scotland, to study national inventories, Class 1 and class 2 peat soils were obtained from the Scottish government and the rest of the classes were excluded for further analysis since they are not defined as ‘priority peatland habitat’.

### Digitalisation of windfarm infrastructures

**Figure 6:**
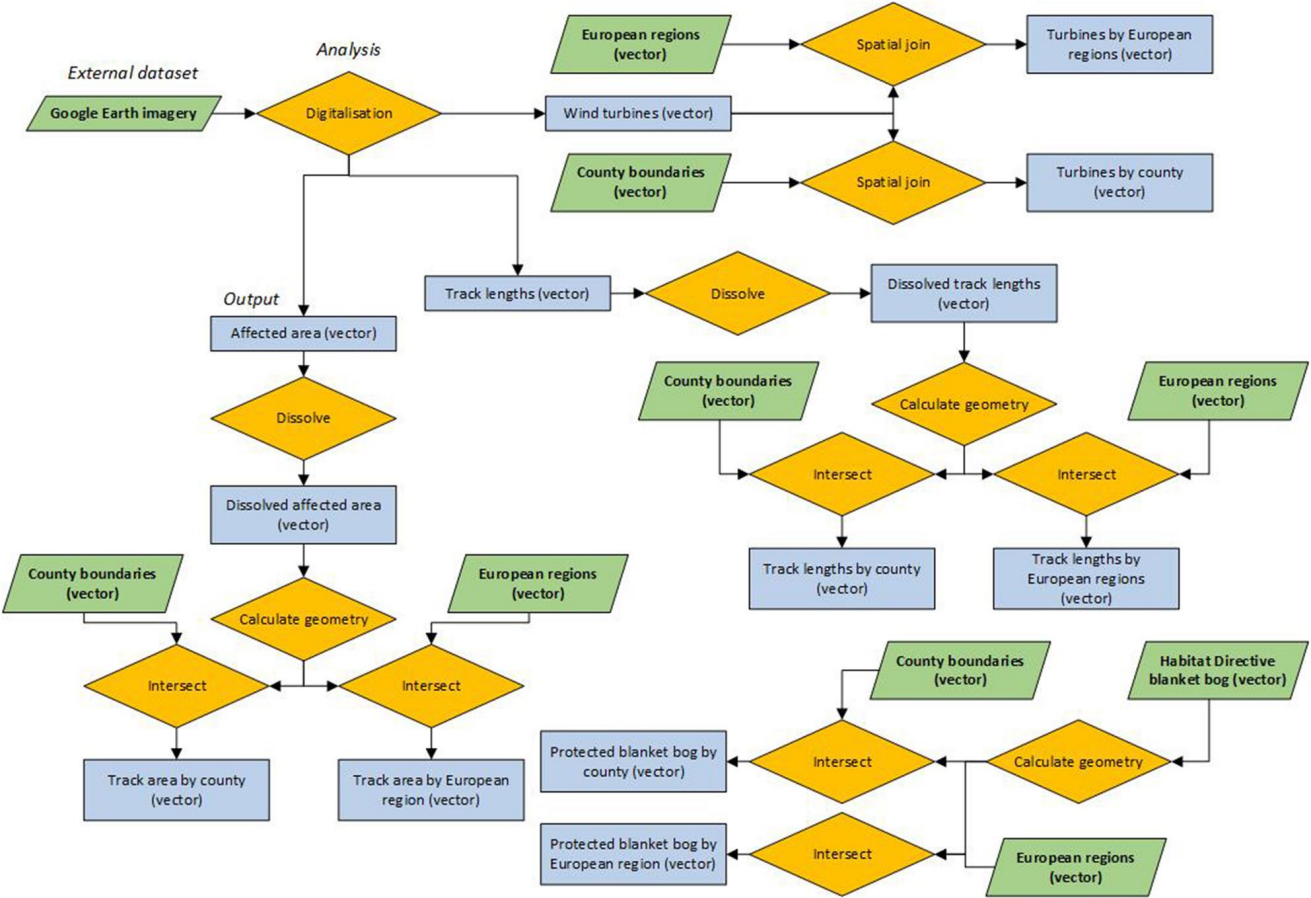
Flowchart of spatial analyses undertook in this research to obtain the final products using ArcGIS 10.8.1 and QGIS.

Google Earth Pro was used to manually digitise current windfarm developments across European blanket bogs. All vector layers obtained from the official government websites were imported into Google Earth Pro and each blanket bog was assessed individually. Individual wind turbines, track length and total area with evidence of windfarm development were digitised using the most recent imagery available at the maximum scale possible to define all the visible infrastructures at the best resolution possible. A file with the current aerial imagery year has been provided in additional materials. All turbines were digitised using a point shapefile, tracks using a polyline shapefile and affected area using a polygon shapefile in ArcGIS 10.8.1 (Fig. 6). All files were exported in .kml format and imported into ArcGIS 10.8.1 for further analysis (Fig. 6). All vector files were managed by country to respect the coordinate systems and geographical projections for each individual region in the analysis.

### Spatial analysis

Once all windfarm infrastructures were digitised, a series of spatial analyses were followed to calculate the number of turbines by European region and counties (Fig. 6). Density estimations were done using ArcGIS 10.8.1 based on the total extent of blanket bog (Fig. 1) by European region and counties, and the total number of wind turbines, track length and total affected areas. The result was multiplied by 10 to reduce decimal numbers and facilitate readability. The density analysis provides a standardised estimation of the total of windfarm infrastructures across blanket bogs assuming they are all equally distributed; however, windfarm developments were usually concentred in more specific areas.

### Statistical analysis

Shapiro normality test was completed in RStudio 1.4 for all variables with recognised blanket bogs studied at the European regional level (Table 5) before undertaking further statistical tests. Since all data was normally distributed, relationships between the recognised area of blanket bogs and the windfarm developments were tested using Pearson correlation test.

**Table 5:** Normality test results of all variables studied at European regions NUTS level 2.

Variable	Test value	*p* value
Recognised blanket bog area	0.44	< 0.001
Total number turbines	0.42	< 0.001
Total length of tracks	0.48	< 0.001
Total affected area	0.50	< 0.001

## Supplementary Information

Supplementary Information.

## Data Availability

The datasets generated and/or analysed during the current study are not publicly available due to restricted access, but are available from the corresponding author on reasonable request.
