# Supplementary material for: The extent of windfarm infrastructures on recognised European blanket bogs
Source: Sci Rep. 2023 Mar 8;13:3919. doi: 10.1038/s41598-023-30752-3 (PMC9995470; doi:10.1038/s41598-023-30752-3)
Supplement: Supplementary file 1 — Supplementary Information. [file 41598_2023_30752_MOESM1_ESM.docx]

**SUMPLEMENTARY INFORMATION**

**THE EXTENT OF WINDFARM infrastructureS ON recognised EUROPEAN blanket bogs**

**Authors**

Chico, G.^1^, Clewer, T.^1^, Midgley, N.G.^1^, Gallego-Anex, P.^1^, Ramil-Rego, P.^2^, Ferreiro, J.^2^, Whayman, E.^1^, Goeckeritz, S.^1^, Stanton, T.^3^

**Authors’ affiliations**

^1^ School of Animal, Rural and Environmental Sciences. Nottingham Trent University, Brackenhurst Campus, Southwell, NG25 0QF, United Kingdom.

^2^ Instituto de Biodiversidade Agraria e Desenvolvemento Rural (IBADER), Universidade de Santiago de Compostela, Campus Terra, 27002, Lugo, Spain

^3^ Department of Geography and Environment, Loughborough University, Loughborough LE11 3TU, United Kingdom

**Table A.** Windfarm developments in relation with the total of turbines and the year commissioned^43–45^.

| **Windfarm** | **Number of turbines** | **Year commissioned** | **Windfarm** | **Number of turbines** | **Year commissioned** |
| --- | --- | --- | --- | --- | --- |
| Drumlough Hill | 1 | 1997 (ext. 2011) | Derrynadivva | 5 | 2009 |
| Lurganboy | 4 | 2008 | Lenanavea | 5 | 2011 |
| Mount Cronalaght | 8 | 1997 | Galway Park | 14 | 2017 |
| Meentycat | 4 | 2004 | Leitir Guingaid | 1 | 2014 |
| Killybegs | 6 | 2009 | Sonnagh Old | 1 | 2004 |
| Barnesmoore | 25 | 1997 | Knocknagoum | 17 | 2013 |
| Meenadreen | 2 | 1999 (ext. 2012) | Pallas I | 3 | 2008 |
| Tullynamoyle | 1 | 2011 (ext. 2017) | Dromadda More | 1 | 2018 |
| Black Banks | 12 | 2001 | Mount Eagle | 2 | 2008 |
| Carranne Hill | 3 | 2006 | Middas | 4 | 2007 |
| Altagowlan | 28 | 2005 | Coomagearlaghy | 6 | 2006 (ext. 2009) |
| Kilronan | 9 | 1997 | Curragh | 8 | 2008 |
| Carrowleagh | 12 | 2012 | Gneeves | 2 | 2005 |
| Corneen | 2 | 2001 | Fiouco | 5 | 2005 |
| Álabe-Cuadramón | 11 | 1999 | Goia-Peñote | 2 | 2005 |
| Álabe-Lomba | 26 | 2001 | Leboreiro | 22 | 2005 |
| Álabe-Nordés | 20 | 1999 | Montouto | 29 | 2001 |
| Álabe-Refachón | 27 | 2001 | Pedra Chantada | 15 | 2003 |
| Álabe-Soán | 24 | 1999 | Pena Grande | 6 | 2003 |
| Álabe-Soán (2) | 23 | 2004 | Pena Luisa | 6 | 2003 |
| Álabe-Ventoada | 19 | 2004 | Rioboo | 5 | 2006 |
| Buio | 4 | 2006 | Silán | 11 | 2003 |

**Table B.** Windfarm developments on recognised blanket bog within a Natura 2000 site in relation with the year commissioned and the designation date.

| **Windfarm** | **Country** | **Year**  **commissioned** | **Natura 2000 site** | **Natura 2000 Code** | **Date**  **designated** |
| --- | --- | --- | --- | --- | --- |
| Knocknagoum | Ireland | 2013 | Stacks to Mullaghareirk Mountains | IE0004161 | 2007 |
| Mount Eagle | Ireland | 2008 | Stacks to Mullaghareirk Mountains | IE0004161 | 2007 |
| Dromadda More | Ireland | 2018 | Stacks to Mullaghareirk Mountains | IE0004161 | 2007 |
| Pallas I | Ireland | 2008 | Stacks to Mullaghareirk Mountains | IE0004161 | 2007 |
| Sonnagh Old | Ireland | 2004 | Slieve Aughty | IE0004168 | 2007 |
| Álabe-Cuadramón | Spain | 1999 | Serra do Xistral | ES1120015 | 1999 |
| Álabe-Lomba | Spain | 2001 | Serra do Xistral | ES1120015 | 1999 |
| Álabe-Nordés | Spain | 1999 | Serra do Xistral | ES1120015 | 1999 |
| Álabe-Refachón | Spain | 2001 | Serra do Xistral | ES1120015 | 1999 |
| Álabe-Soán | Spain | 1999 | Serra do Xistral | ES1120015 | 1999 |
| Álabe-Soán Ampl. | Spain | 2004 | Serra do Xistral | ES1120015 | 1999 |
| Álabe-Ventoada | Spain | 2004 | Serra do Xistral | ES1120015 | 1999 |
| Buio | Spain | 2006 | Serra do Xistral | ES1120015 | 1999 |
| Fiouco | Spain | 2005 | Serra do Xistral | ES1120015 | 1999 |
| Leboreiro | Spain | 2005 | Serra do Xistral | ES1120015 | 1999 |
| Montouto | Spain | 2001 | Serra do Xistral | ES1120015 | 1999 |
| Pedra Chantada | Spain | 2003 | Serra do Xistral | ES1120015 | 1999 |
| Pena Grande | Spain | 2003 | Serra do Xistral | ES1120015 | 1999 |
| Pena Luisa | Spain | 2003 | Serra do Xistral | ES1120015 | 1999 |
| Rioboo | Spain | 2006 | Serra do Xistral | ES1120015 | 1999 |
| Silán | Spain | 2003 | Serra do Xistral | ES1120015 | 1999 |
